# Are Total, Intensity- and Domain-Specific Physical Activity Levels Associated with Life Satisfaction among University Students?

**DOI:** 10.1371/journal.pone.0118137

**Published:** 2015-02-19

**Authors:** Željko Pedišić, Zrinka Greblo, Philayrath Phongsavan, Karen Milton, Adrian E. Bauman

**Affiliations:** 1 Faculty of Kinesiology, University of Zagreb, Zagreb, Croatia; 2 Prevention Research Collaboration, Sydney School of Public Health, The University of Sydney, Sydney, Australia; 3 Department of Psychology, Centre for Croatian Studies, University of Zagreb, Zagreb, Croatia; Universidad de Granada, SPAIN

## Abstract

**Background:**

Thorough information about the relationship between physical activity (PA) and life satisfaction is still lacking. Therefore, this study examined the cross-sectional relationships between life satisfaction and meeting the World Health Organization (WHO) moderate to vigorous-intensity PA recommendations, total volume and duration of PA, intensity-specific PA (walking, moderate- and vigorous-intensity), domain-specific PA (work, transport-related, domestic, and leisure-time), and 11 domain and intensity-specific PA types among university students. Additionally, we examined the associations between life satisfaction and gender, age, disposable income, community size, smoking, alcohol intake, body mass index (BMI), and self-rated health.

**Methods:**

The study included a random sample of 1750 university students in Zagreb, Croatia (response rate = 71.7%; 62.4% females; mean age 21.5 ± 1.8 years), using the International Physical Activity Questionnaire — long form and the Satisfaction with Life Scale.

**Results:**

Higher life satisfaction was associated with female gender (*β* = 0.13; *p* = <0.001), younger age (*β* = -0.07; *p* = 0.024), higher disposable income (*β* = 0.10; *p* = 0.001), and better self-rated health (*β* = 0.30; *p* = <0.001). No significant association was found between life satisfaction and size of community (*p* = 0.567), smoking status (*p* = 0.056), alcohol consumption (*p* = 0.058), or BMI (*p* = 0.508). Among all PA variables, only leisure-time vigorous-intensity PA was significantly associated with life satisfaction after adjustments for socio-demographic characteristics, lifestyle and self-rated general health (*β* = 0.06; *p* = 0.045).

**Conclusions:**

This study indicated a weak positive relationship between leisure-time vigorous-intensity PA and life satisfaction, whilst no such association was found for other PA variables. These findings underscore the importance of analyzing domain and intensity-specific PA levels in future studies among university students, as drawing conclusions about the relationship between PA and life satisfaction based on total PA levels only may be misleading.

## Introduction

### Life satisfaction

Life satisfaction is defined as an overall cognitive and judgmental assessment of personal quality of life according to self-selected criteria [1]. Although personal satisfaction may vary across specific life domains, most previous studies have focused on global life satisfaction; defined as a unidimensional characteristic [2]. Life satisfaction is a relatively stable characteristic, more tied to systematic and chronic factors, than to temporary and transitory affective states [3]. It has been shown that cognitive judgment of personal life satisfaction is an essential component of subjective well-being and a valid indicator of overall life quality [4]. As such, life satisfaction is one of the central constructs in the field of positive psychology [5]. Prospective studies suggest that lower life satisfaction is associated with an increased risk of disease-related, injury-related, and all-cause mortality [6–8], suicidal behaviour [9], major weight gain [10], and moderate or severe depression [11]. It has been suggested that low life satisfaction may negatively affect subsequent income, employment and marital status [12]. Furthermore, cross-sectional studies have shown that poor life satisfaction is associated with a range of unhealthy behaviours [13,14] and youth violence [15].

Average life satisfaction among adults across nations ranges between 60% and 80% of its maximum score and significantly varies between individuals [16,17]. Interestingly, more than 75% of young adults are not completely satisfied with their lives [14]. An international study has shown that the average life satisfaction of adolescents varies between ~55% and 75% of its maximum score across different nations [18]. Among children from 36 countries the mean life satisfaction score was found to be 76% of its maximum [19]. Although most children rate their lives positively, there is still a significant proportion who report very low levels of life satisfaction [2]. To effectively identify and influence individuals and groups at the highest risk, public health strategies need to be informed by empirical evidence about the determinants of life satisfaction. Previous studies have investigated the demographic, psychological, social, environmental, cultural, behavioural and health-related determinants of life satisfaction [5,20].

Furthermore, different theories have been developed to explain the determinants of life satisfaction. *Activity theory* suggests that life satisfaction is determined by the frequency of participation in given activities and the degree of intimacy associated with the activities [21]. Although the theory was originally developed to help explain the process of successful aging, its premises regarding life satisfaction have also been confirmed among a general adult population [22]. Interestingly, in an empirical examination of *activity theory* Longino and Kart [23] found that life satisfaction is: 1) positively affected by participation in informal activities, 2) not impacted by participation in solitary activities, and 3) negatively affected by participation in formal activities. *Need theory* implies that life satisfaction is mostly regulated by the ability of an individual to satisfy his/her biological and psychological needs [24]. Rodriguez et al. [22] suggested that ‘fulfilling needs’ is a better predictor of life satisfaction than ‘participation in activities’. Their study identified ‘satisfying the social need’ and ‘satisfying the autonomy need’ as the strongest predictors of life satisfaction.

### Relationship between physical activity and life satisfaction

It has been shown that regular physical activity (PA) may be associated with increased life satisfaction among all age groups: children and adolescents [5,25–30]; young adults [14,31–37]; adults [38–43]; and elderly [44–48]. Life satisfaction has been shown to be positively related to overall leisure-time PA [31,34,37,39,42], participation in sports/exercise [14,22,25–30,32,35,38,41,43,44], and participation in several specific types of PA, such as stretching [26], strength training [22,26], jogging [39], and walking [44,45,48]. By contrast, mixed results were observed for non-domain-specific PA (sum of PA levels across two or more domains), indicating its positive [36,37,40,46] or non-significant [33,36,37,48,49] relationship with life satisfaction. Interestingly, a recent study separately examined PA in sporting clubs, gymnasia, and walking among Australian rural-living women and found a significant association with life satisfaction only for type, but not for level of PA [50].

### Distinct effects of domain and intensity-specific PA levels

The World Health Organization (WHO) suggests that moderate or vigorous-intensity PA (MVPA) required for health benefits can be accumulated in any domain—work, domestic, transport, or leisure-time [51]. However, recent studies have shown that different domains of PA may have distinct associations with health-related and overall quality of life [52–56], symptoms of depression [57–59], symptoms of anxiety [60] and all-cause mortality [61]. For example, a study by Jurakic et al. [52] has shown that leisure-time PA may be positively, whilst transport-related and domestic PA are inversely related to health-related quality of life. In another study, lower depression was associated with higher levels of leisure-time PA and lower levels of occupational PA [58]. Furthermore, the magnitude and direction of the relationships between different domains of PA and health outcomes may be moderated by age and gender [52,53,55]. For instance, Peeters et al. [55] found that higher levels of domestic PA are associated with increased well-being among mid-aged and older women and with decreased well-being among young women. Cerin et al. [53] found no association between household and occupational PA and self-perceived mental health among men, but they observed a negative relationship between these variables among women. Furthermore, previous studies have also indicated that different intensity-levels (e.g. moderate-intensity, vigorous-intensity) may have distinct relationships with mental health outcomes [53,57]. For example, self-perceived mental health was positively related to vigorous-intensity leisure-time PA, whilst it showed no significant relationship with moderate-intensity PA in the same domain [53]. It seems, therefore, that non-domain-specific and non-intensity specific PA guidelines may not be most effective for achieving all psychological benefits. The emerging evidence about differential effects of domain and intensity-specific PA calls for further research, particularly regarding psychological outcomes that have not been studied in this context.

Many studies examining PA and life satisfaction have assessed physical exercise or sports participation as specific types of leisure-time activity [14,22,25–30,32,33,35,38,41,43,44,62]. Several studies assessed overall leisure-time PA [31,34,37,39,42] or non-domain-specific PA [33,36,37,40,46,48,49]. Specific types of PA were separately analysed by Valois et al. [26] (walking/riding a bike), Morgan and Bath [45] (walking), and Withall et al. [48] (total number of steps taken). Morgan and Bath [45] also separately assessed PA level in the domestic domain. This shows that most previous studies on the relationship between PA and life satisfaction have focused on total PA and the leisure-time domain, while evidence for work, domestic, and transport domains is scarce. Moreover, none of the studies assessed all PA domains (work, transport, domestic, leisure-time) separately. Furthermore, most studies used single-items or short questionnaires which did not allow to separately examine different intensities (low, moderate, vigorous) or types of PA (e.g., walking, cycling). Although few studies used instruments that would allow them to separately analyse different intensity levels, such as the International PA Questionnaire—Short form (IPAQ-short) [36,37] and accelerometers [36,48], none of them reported separately on intensity-specific PA levels. In addition, none of the studies used instruments that would allow for separate assessment of intensity levels within specific domains of PA, i.e. domain and intensity-specific PA (e.g. vigorous-intensity PA in the work domain, moderate-intensity PA in the work domain, vigorous-intensity PA in the domestic domain, moderate-intensity PA in the domestic domain). Therefore, thorough information about the relationship between life satisfaction and domain and intensity-specific PA levels is still lacking.

### Aims and hypotheses

**Fig 1:**
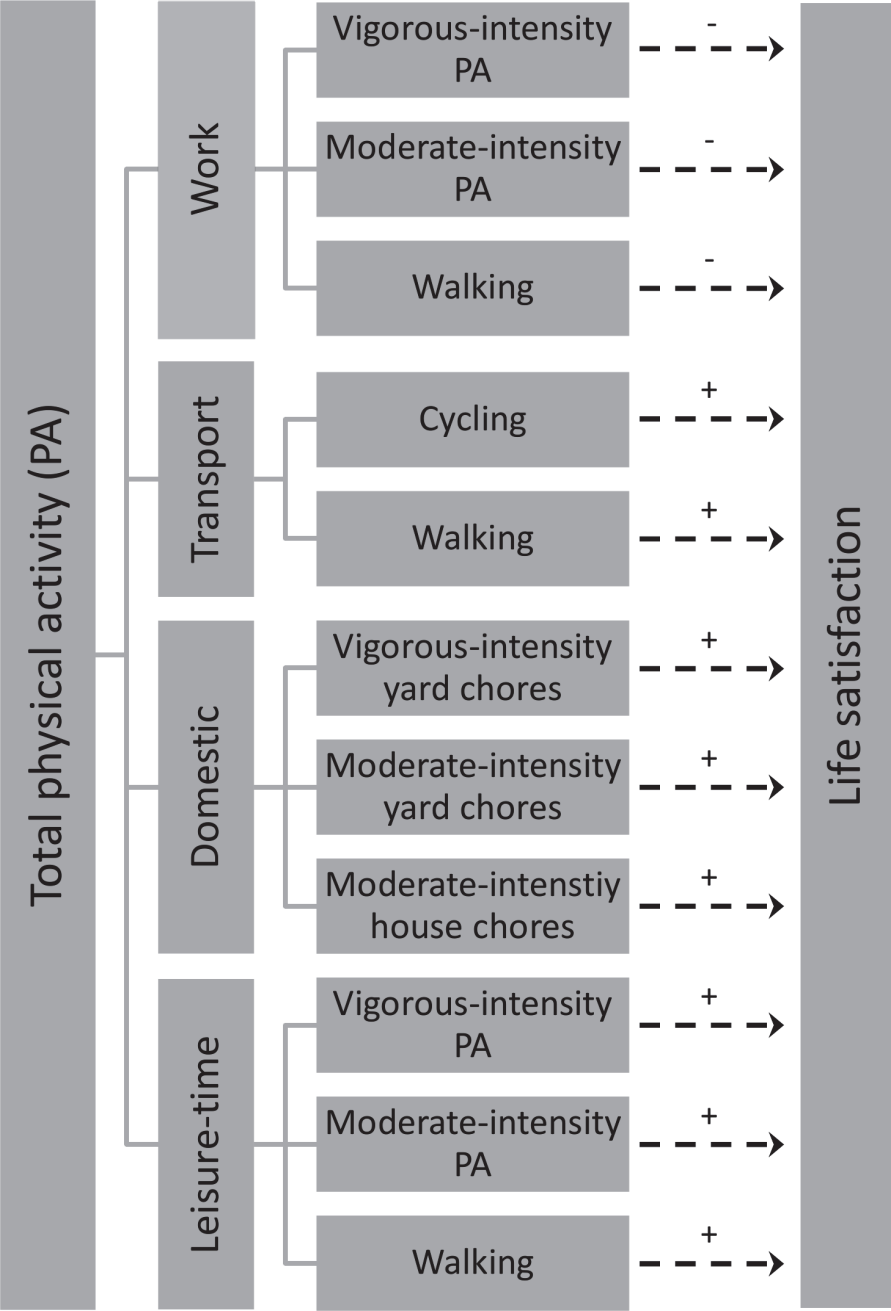
Hypothesised relationships between domain and intensity-specific physical activity levels and life satisfaction. *Right dashed arrow*: hypothesized relationship tested in the present study. *Sign above the arrow*: hypothesized direction of the relationship (‘+’ = positive; ‘-’ = inverse).

The magnitude and direction of the relationship between PA and life satisfaction may be dependent on the study population and instruments used. In order to compare the relative contributions of different domain and intensity-specific PA levels to life satisfaction, it is essential to assess them all within a single study. Besides, studies have shown that age can act as a moderator of the determinants of life satisfaction [63], which indicates the importance of research within specific age groups. Hence, the aim of this study was to examine the relationship between life satisfaction and intensity-specific PA levels in work, domestic, transport and leisure-time domains among university students. Better understanding of this relationship may increase the public health utility of PA intervention strategies to influence life satisfaction among university students, and help elucidate whether non-domain and non-intensity specific PA recommendations are justified in such interventions. The hypothesized relationships are presented in Fig. 1.

The hypotheses were proposed based on: 1) *activity theory*, 2) *need theory*, and 3) previous empirical findings.

Hypothesis 1: Leisure-time PA is positively associated with life satisfaction

The positive relationship between leisure-time PA and life satisfaction was hypothesized for several reasons. Firstly, we assumed that among young adults the degree of intimacy associated with leisure-time PA is higher than with activities in other domains. Secondly, participation in leisure-time PA may satisfy the social need, autonomy need, and physical fitness need, that are shown to be among strongest predictors of life satisfaction [22]. Thirdly, several previous studies have found a positive relationship between leisure-time PA and life satisfaction among young adults [14,31,32,34,35,37].

Hypothesis 2: Domestic and transport-related PA levels are positively associated with life satisfaction

We assumed that domestic and transport-related activities are mostly solitary for young adults. As such, according to Longino and Kart [23], they would not be expected to show significant association with life satisfaction. Accordingly, it can be assumed that these activities have very little capacity to contribute in satisfying the social need of young adults. Jurakic et al. [52] suggested that some people may even perceive the participation in these activities as a restriction on their autonomy. Some people may participate in such activities because they do not have an alternative (e.g. they have to cycle to work, because they cannot afford to buy a car). This may suggest that according to the *need theory* these activities are not likely to increase life satisfaction. Despite that, previous studies have indicated that participating in domestic PA, such as gardening and housework, may be positively related to life satisfaction [45,64,65]. Furthermore, a recent review suggested that active transport has potential to indirectly increase subjective well-being through its positive impact on travel satisfaction [66]. It therefore seems possible that these domains of PA are linked to life satisfaction through mechanisms that are not explained by *activity theory* and *need theory*. Hence, based on the recent empirical findings, we hypothesized a positive relationship between life satisfaction and domestic and transport-related PA.

Hypothesis 3: Occupational PA is negatively associated with life satisfaction

We assumed that for young people occupational activities are contextually most similar to formal activities described by Longino and Kart [23], which would suggest that they are likely to be inversely related to life satisfaction. It should be noted, however, that this assumption may equally relate to participation in both sedentary and physically demanding occupational activities. Furthermore, a study has shown that a high physical work load is associated with low job control [67]. We therefore assumed that physically demanding occupational activities may reduce young people’s self-perceived autonomy by imposing restrictions and control, and consequently negatively affect their life satisfaction. Furthermore, previous findings about the relationship between occupational PA and psychological health and well-being were equivocal. For example, Laaksonen et al. [67] have shown that higher physical work load is associated with poor self-reported mental health among middle-aged employees, whilst several other studies found no relationship between occupational PA and overall and health-related quality of life [52–54,56]. Despite these equivocal findings, based on the above-mentioned assumptions from the *activity theory* and *need theory*, we hypothesized a negative relationship between occupational PA and life satisfaction.

Hypothesis 4: The strength of the relationship between PA and life satisfaction increases with increased intensity of PA

When compared to moderate-intensity and walking, vigorous-intensity PA has shown stronger associations with self-reported mental health (among men) [68], mental well-being (among both genders) [53], and symptoms of depression, anxiety and somatisation (among men) [69]. We assumed that the effect sizes of the relationships between intensity-specific PA levels and life satisfaction may follow a similar pattern.

Additionally, we examined the associations between life satisfaction and: 1) meeting the WHO MVPA recommendations; 2) total (i.e. non-domain-specific and non-intensity specific) PA level; 3) gender; 4) age; 5) disposable income; 6) community size; 7) smoking; 9) alcohol intake; 10) body mass index (BMI); and 11) self-rated health.

## Materials and Methods

### Participants

The study was carried out in a stratified random sample of students living in the university residence halls in Zagreb, Croatia. A two-stage stratification was performed according to gender and all five university residence halls in Zagreb. During the second week of June 2009, 1750 students were invited by mail to take part in the study. A week later a second invitation was sent to the students who did not respond initially. In total, 71.7% of invited students consented to participate (*n* = 1254), and were surveyed in the official premises of their resident halls. Eight trained surveyors participated in the process of data collection. The survey took place during the exams period. The sample included students from all 29 faculties of the University of Zagreb and 7 private colleges. With approximately 70,000 students enrolled in its programs, the University of Zagreb is the largest university in Croatia and one of the largest higher education institutions in the European Union. The random sample covered students from all major academic disciplines in natural sciences, social sciences, arts and humanities, formal sciences, and applied sciences and professions. A total of 1163 participants (62.4% females; mean ± standard deviation age, 21.5 ± 1.8 years; 2.6% enrolled in physical education or sport science academic degrees) with data on PA comprised the final analytic sample. A post-hoc power analysis indicated that the sample size was sufficient to detect a significant multiple correlation coefficient at *p* < 0.05 with the power of 85%, if the population effect size was at least small (Cohen *f* ^*2*^ ≥ 0.02) [70]. Participation was voluntary and all participants gave written informed consent before entering the study. The study was approved by the Institutional Review Board at the Faculty of Kinesiology, University of Zagreb. Socio-demographic and other relevant characteristics of the sample are presented in Table 1.

**Table 1:** Sample characteristics (n = 1163).

Variable / category	Mean ± SD^a^
Age (years)	21.5 ± 1.8
Disposable income (€)	212 ± 104
Life satisfaction (5–25)	17.9 ± 3.3
Physical activity (minutes/week)	**Median (IQR)** ^b^
Vigorous-intensity at work	0 (0)
Moderate-intensity at work	0 (0)
Walking at work	0 (0)
Cycling for transport	0 (0)
Walking for transport	210 (330)
Vigorous-intensity yard chores	0 (0)
Moderate-intensity yard chores	20 (120)
Moderate-intensity house chores	60 (120)
Vigorous-intensity in leisure-time	0 (120)
Moderate-intensity in leisure-time	0 (30)
Walking in leisure-time	90 (210)
Work domain	0 (0)
Transport domain	235 (320)
Domestic domain	120 (310)
Leisure-time domain	210 (360)
Vigorous-intensity	0 (135)
Moderate-intensity	210 (420)
Walking	390 (620)
Total (minutes/week)	780 (1025)
Total (MET-minutes/week)	3133 (4122)
Gender	%
Female	62.4
Male	37.6
**Size of community** ^c^	
< 2000	22.7
2001–10 000	32.8
10 001–100 000	34.4
> 100 000	10.0
**Meeting MVPA recommendation** ^d^	
Standard^e^	71.5
For additional benefits^f^	58.5
Smoking status	
never smoked	70.0
quit smoking	8.1
smoking <5 cigarettes/day	7.4
smoking 5–10 cigarettes/day	5.9
smoking 10–20 cigarettes/day	6.8
smoking >20 cigarettes/day	1.8
Alcohol intake	
never	20.2
<1 day/week	56.9
1–3 days/week	21.6
4–6 days/week	1.0
everyday	0.2
**Body mass index (kg/m** ^**2**^ **)** ^g^	
< 18.5	6.2
18.5–25	79.7
25–30	12.5
≥ 30	1.6
**Self-rated general health** ^h^	
Poor	1.3
Fair	12.2
Good	20.1
Very Good	46.2
Excellent	20.1

^a^Mean ± standard deviation.

^b^Median (interquartile range).

^c^Number of inhabitants of the city/town/village of origin.

^d^World Health Organization recommendation for moderate to vigorous-intensity physical activity.

^e^≥75 min/week of vigorous-intensity or ≥150 min/week of moderate-intensity physical activity.

^f^≥150 min/week of vigorous-intensity or ≥300 min/week of moderate-intensity physical activity.

^g^Calculated from self-reported height and weight.

^h^Single-item: “In general, how would you rate your health?”

### Instruments

PA levels were assessed using the International Physical Activity Questionnaire (IPAQ)—long form [71]. The questionnaire consists of 27 items about the frequency and usual duration of PA and sedentary behaviour in the last 7 days. It provides intensity-specific data on PA in the domains of: 1) work (vigorous intensity, moderate intensity, and walking); 2) transport (walking and cycling); 3) domestic and garden (vigorous-intensity yard chores, moderate-intensity yard chores, and moderate-intensity household chores); and 4) leisure-time (vigorous intensity, moderate intensity, and walking). In the present study, the domain and intensity-specific PA levels were expressed in minutes per week. Intensity-specific scores were calculated by summing all the minutes spent: 1) walking; 2) in moderate-intensity PA; and 3) in vigorous-intensity PA. Domain-specific scores were calculated by summing all the minutes spent in: 1) work-related PA; 2) transport-related PA; 3) domestic PA; and 4) leisure-time PA, regardless of their intensity. The total PA score was calculated as a sum of all domain and intensity specific PA minutes weighted by their metabolic equivalents (METs) and expressed in MET-minutes per week, according to the official IPAQ data processing guidelines [72]. Additionally, we calculated the total time spent in PA as a simple sum (non-intensity-weighted) of all domain and intensity specific PA minutes. The achievement of the WHO recommendations in terms of: 1) standard MVPA recommendation (≥ 75 minutes/week of vigorous-intensity PA or ≥ 150 minutes/week of moderate-intensity PA or an equivalent combination of both); and 2) MVPA recommendation for additional health benefits (≥ 150 minutes/week of vigorous-intensity PA or ≥ 300 minutes/week of moderate-intensity PA or an equivalent combination of both) [51], was also calculated from the IPAQ data. Previous studies have demonstrated good measurement properties of the IPAQ—long form [71,73]. A detailed description of the questionnaire can be found elsewhere [72].

Life satisfaction was measured using the Satisfaction With Life Scale (SWLS) [1]. The scale consists of 5 statements (for example, “In most ways my life is close to my ideal.”) and participants rated their agreement or disagreement with each statement using a 5-point Likert scale. Overall life satisfaction (on the scale from 5–25) was calculated as the sum of responses to all items, where a higher score indicated a higher level of life satisfaction. Previous studies have consistently demonstrated high reliability and a single-factor structure of SWLS items [74,75].

A separate set of questions asked about: gender; age; size of community, i.e. the number of inhabitants of city/town/village of participant’s origin ({1} < 2000, {2} 2001–10 000, {3} 10 001–100 000, and {4} > 100 000); disposable income; smoking status ({1} never smoked, {2} quit smoking, {3} smoking <5 cigarettes/day, {4} smoking 5–10 cigarettes/day, {5} smoking 10–20 cigarettes/day, or {6} smoking >20 cigarettes/day); alcohol intake ({1} never, {2} <1 day/week, {3} 1–3 days/week, {4} 4–6 days/week, {5} everyday); self-reported height and weight (used to compute body mass index (BMI; kg/m^2^); and self-rated general health (single item “In general, how would you rate your health?” with the following response scale: {1} Poor, {2} Fair, {3} Good, {4} Very Good, and {5} Excellent).

### Data analysis

The data on socio-demographic and lifestyle characteristics, life satisfaction, and self-rated health were presented as mean ± standard deviation for quantitative variables, and percentages for qualitative variables. Due to their non-normal distribution, the continuous PA data were presented using medians and interquartile ranges, which is in accordance with the official guidelines for data processing and analysis of the IPAQ [72]. The relationship between PA behaviour (independent variables) and life satisfaction (dependent variable) was assessed by 7 separate linear regression models. The independent variables in the regression models were: (i) meeting the WHO standard MVPA guidelines (Model 1); (ii) meeting the WHO MVPA guidelines for additional health benefits (Model 2); (iii) total volume of PA expressed in MET-hours/day (Model 3); (iv) total duration of PA expressed in hours/day (Model 4); (v) intensity-specific PA levels (vigorous-intensity, moderate-intensity, and walking) (Model 5); (vi) domain-specific PA levels (work, transport-related, domestic, and leisure-time) (Model 6); and (vii) domain and intensity-specific PA levels (vigorous-intensity at work, moderate-intensity at work, walking at work, cycling for transport, walking for transport, vigorous-intensity yard chores, moderate-intensity yard chores, moderate-intensity house chores, vigorous-intensity in leisure-time, moderate-intensity in leisure-time, and walking in leisure-time) (Model 7), whilst the total SWLS score was used as the dependent variable in all models. In the first step, Models 1–4 and Models 5–7 were analysed using the simple linear regression (with no adjustments) and the multiple linear regression (with mutual adjustments of PA variables within the model), respectively. In the second step, the regression models were additionally adjusted for gender, age, size of community, disposable income, smoking status, alcohol intake, BMI and self-rated health, because previous studies have indicated that these variables could act as confounders of the proposed relationship [63,76–79]. Gender was entered to the model as a dichotomous variable, size of community, smoking status, alcohol intake and self-rated health as ordinal variables, and age, disposable income and BMI as continuous variables. Prior to the regression analysis, reported alcohol intakes on ‘4–6 days/week’ and ‘everyday’ were collapsed into a single category, due to the low number of such responses. Standardized and unstandardized regression coefficients, partial correlations, and coefficients of multiple determination were calculated and tested for statistical significance. The unstandardized regression coefficients for continuous PA variables were presented in hours/day instead of minutes/week. The partial correlations were interpreted as a measure of relative effect size, and classified as: small (0.10–0.29); medium (0.30–0.49); and large (≥0.50), according to Cohen [70]. Further, a previous study has shown that gender may act as a moderator of the relationship between PA and mental well-being [53]. Therefore, using the General Linear Models (GLM) procedure we tested all the unadjusted and adjusted models for the possible interaction effect between gender and PA variables. Prior to the analysis, all regression models were inspected for the normality of residuals, linearity of relationships, multicollinearity, and heteroscedasticity using the normal probability plot, bivariate scatterplot of each independent variable vs. residuals, variance inflation factors, and predicted vs. residuals plot, respectively. All analyses were performed using IBM SPSS Statistics 21 (SPSS Inc. an IBM Company, Chicago, IL, USA), with the level of statistical significance set at *p* < 0.05.

## Results

Most students (67.2%) originated from cities and towns with a population of 2001–100,000. The prevalence of regular alcohol consumption (≥ once a week) and smoking was 22.5% and 21.9%, respectively. According to their BMI, approximately 14% of the students were classified as overweight or obese, and 6.2% as underweight. The majority of students rated their general health as ‘very good’ or ‘excellent’ (66.5%). The mean (± standard deviation) life satisfaction score in our sample was 17.9 ± 3.3, that is, 64.5% of the maximum possible score. The life satisfaction score was below 80.0% of its maximum in 80.1% of the participants and below 50.0% of its maximum in 14.0% of the participants. Walking for transport was the type of PA in which the participants, on average, spent the greatest amount of time (median = 210 minutes/week), followed by walking for leisure (median = 90 minutes/week), and moderate-intensity chores in the household (median = 60 minutes/week) and yard (median = 20 minutes/week). The majority of participants did not report spending any time in other domain and intensity-specific types of PA. No vigorous-intensity PA at work was reported by 92.9%, no moderate-intensity PA at work by 91.4%, no walking at work by 88.9%, no transport-related cycling by 87.0%, no vigorous-intensity yard chores by 83.6%, no vigorous-intensity leisure-time PA by 55.2%, and no moderate-intensity leisure-time PA by 72.1% participants (S1 Table). Overall, participants reported spending the greatest amount of time in transport-related PA (median = 235 minutes/week), followed by leisure-time (median = 210 minutes/week) and domestic activities (median = 120 minutes/week). The majority of participants (85.1%) did not report spending any time in work-related PA. Furthermore, median was 390 minutes/week and 210 minutes/week for walking and moderate-intensity PA, respectively, whilst slightly more than half of the participants (51.5%) did not report any participation in vigorous-intensity PA. Medians of 780 minutes/week and 3133 MET-minutes/week were observed for the total PA level.

Variance inflation factors for the independent variables in the regression models ranged from 1.00 to 1.76 indicating no severe multicollinearity. The regression assumptions of normality, linearity and homoscedasticity were also met. Prior to the main analysis, life satisfaction was regressed on the potential confounding variables (socio-demographic and lifestyle characteristics and self-rated health; Table 2). Higher life satisfaction scores were associated with female gender (*β* = 0.13; *p* = <0.001), younger age (*β* = -0.07; *p* = 0.024), higher disposable income (*β* = 0.10; *p* = 0.001), and better self-rated health (*β* = 0.30; *p* = <0.001). No significant association was found between life satisfaction and size of community (*p* = 0.567), smoking status (*p* = 0.056), alcohol intake (*p* = 0.058), and body mass index (*p* = 0.508). In a sensitivity analysis, size of community, smoking status, alcohol intake, body mass index and self-rated health were entered to the model as categorical variables, but no interpretative differences were observed when compared to the above-mentioned results (data not shown).

**Table 2:** Relationship between socio-demographic variables, lifestyle characteristics and self-rated general health and life satisfaction.

Independent variable	*β* ^a^	*B* ^b^	*r*_XY∙Z_ ^c^	*p* ^d^
Gender (0 = male; 1 = female)	0.13	0.87	0.11	<0.001
Age (years)	-0.07	-0.12	-0.07	0.024
**Size of community** ^e^	0.02	0.06	0.02	0.567
Disposable income (€)	0.10	0.00	0.10	0.001
**Smoking status** ^f^	-0.06	-0.14	-0.06	0.056
**Alcohol intake** ^g^	0.06	0.28	0.06	0.058
Body mass index (kg/m^2^)	0.02	0.03	0.02	0.508
**Self-rated general health** ^h^	0.30	1.03	0.29	<0.001
**Multiple R-square** ^i^	0.12			<0.001

^a^Standardized regression coefficient.

^b^Unstandardized regression coefficient.

^c^Partial correlation.

^d^p-value for regression coefficients.

^e^Population of city/town/village of participant’s origin categorized as: [1] < 2000, [2] 2001–10 000, [3] 10 001–100 000, and [4] > 100 000.

^f^Smoking status categorized as: [1] never smoked, [2] quit smoking, [3] smoking <5 cigarettes/day, [4] smoking 5–10 cigarettes/day, [5] smoking 10–20 cigarettes/day, and [6] smoking >20 cigarettes/day.

^g^Alcohol intake categorized as: [1] never, [2] <1 day/week, [3] 1–3 days/week, and [4] 4 or more days/week.

^h^Self-rated general health categorized as: [1] Poor, [2] Fair, [3] Good, [4] Very Good, and [5] Excellent.

^i^Coefficient of multiple determination.

In the unadjusted models, higher life satisfaction was significantly associated with meeting standard (*β* = 0.07; *p* = 0.024) and additional (*β* = 0.07; *p* = 0.022) MVPA recommendations (Table 3). In the unadjusted models, positive relationships with life satisfaction were also found for the total volume (*β* = 0.07; *p* = 0.013) and total duration (*β* = 0.06; *p* = 0.029) of PA. In the unadjusted analysis of intensity-specific PA levels, significant relationships with life satisfaction were determined for vigorous-intensity (*β* = 0.08; *p* = 0.010) and walking (*β* = 0.06; *p* = 0.046). In the unadjusted analysis of domain-specific PA levels, the only significant relationship with life satisfaction was found for the leisure-time domain (*β* = 0.08; *p* = 0.012). Significant multiple correlation coefficients were found for both the intensity-specific and domain-specific models (Models 5 and 6). However, all the above-mentioned associations became non-significant after their further adjustments for socio-demographic characteristics, lifestyle and self-rated general health. Furthermore, leisure-time vigorous-intensity PA was the only domain and intensity-specific variable to show a significant relationship with life satisfaction. The significant relationship was found in both unadjusted (*β* = 0.10; *p* = 0.001) and adjusted (*β* = 0.06; *p* = 0.045) models. Participation in higher levels of leisure-time vigorous-intensity PA by an hour per day was associated with an average increase in life satisfaction of 0.49 points, which is 2.5% of its total range or 14.8% of its standard deviation. This variable accounted for 1.0% and 0.36% of the life satisfaction variance in the unadjusted and adjusted models, respectively. Time spent in other domain and intensity-specific types of PA was not significantly related to life satisfaction. In both unadjusted and adjusted domain and intensity-specific models (Model 7), the multiple correlation coefficients were not significant.

**Table 3:** Relationship between total, domain- and intensity-specific physical activity levels (independent variables) and life satisfaction (dependent variable) in university students.

Physical activity variable	Unadjusted^a^				Adjusted^b^			
	*β* ^c^	*B* ^d^	*r*_XY∙Z_ ^e^	*p* ^f^	*β* ^c^	*B* ^d^	*r*_XY∙Z_ ^e^	*p* ^f^
Model 1 (meeting guidelines)								
Meeting standard MVPA recommendation (yes = 1; no = 2)^g^	0.07	0.49	0.07	0.024	0.04	0.31	0.04	0.143
Model 2 (meeting guidelines)								
Meeting additional MVPA recommendation (yes = 1; no = 2)^h^	0.07	0.45	0.07	0.022	0.04	0.25	0.04	0.191
Model 3 (total volume)								
Total (MET-hours/day)	0.07	0.03	0.07	0.013	0.04	0.02	0.05	0.114
Model 4 (total duration)								
Total (hours/day)	0.06	0.11	0.06	0.029	0.04	0.06	0.04	0.184
**Model 5 (intensity-specific)** ^i^								
Vigorous-intensity	0.08	0.50	0.08	0.010	0.05	0.34	0.05	0.085
Moderate-intensity	-0.01	-0.04	-0.01	0.657	-0.02	-0.04	-0.02	0.604
Walking	0.06	0.17	0.06	0.046	0.04	0.11	0.04	0.176
**Multiple R-square** ^j^	0.01			0.010	0.00			0.161
**Model 6 (domain-specific)** ^i^								
Work domain	-0.01	-0.03	-0.01	0.783	0.00	-0.01	0.00	0.914
Transport domain	0.03	0.11	0.03	0.375	0.02	0.06	0.02	0.586
Domestic domain	0.00	0.01	0.00	0.955	-0.01	-0.03	-0.01	0.778
Leisure-time domain	0.08	0.30	0.07	0.012	0.05	0.21	0.05	0.081
**Multiple R-square** ^j^	0.01			0.047	0.00			0.358
**Model 7 (domain and intensity specific)** ^i^								
Vigorous-intensity at work	-0.02	-0.18	-0.01	0.632	-0.01	-0.08	-0.01	0.838
Moderate-intensity at work	-0.02	-0.18	-0.02	0.449	-0.04	-0.28	-0.04	0.226
Walking at work	0.04	0.33	0.03	0.266	0.04	0.41	0.04	0.157
Cycling for transport	-0.03	-0.32	-0.03	0.384	0.00	-0.03	0.00	0.932
Walking for transport	0.05	0.20	0.04	0.133	0.02	0.09	0.02	0.479
Vigorous-intensity yard chores	0.01	0.12	0.01	0.683	0.01	0.10	0.01	0.746
Moderate-intensity yard chores	0.02	0.13	0.01	0.657	0.02	0.15	0.02	0.594
Moderate-intensity house chores	-0.02	-0.14	-0.02	0.585	-0.03	-0.23	-0.03	0.347
Vigorous-intensity in leisure-time	0.10	0.79	0.10	0.001	0.06	0.49	0.06	0.045
Moderate-intensity in leisure-time	0.00	0.03	0.00	0.930	0.01	0.08	0.01	0.809
Walking in leisure-time	0.01	0.08	0.01	0.666	0.01	0.08	0.01	0.669
**Multiple R-square** ^j^	0.02			0.064	0.01			0.531

^a^Models 1-4-unadjusted; models 5-7-adjusted for other physical activity variables in the model.

^b^Additionally adjusted for gender, age, community size, disposable income, body mass index, smoking status, alcohol consumption, and self-rated general health.

^c^Standardized regression coefficient.

^d^Unstandardized regression coefficient.

^e^Partial correlation.

^f^p-value for regression coefficients.

^g^≥75 min/week of vigorous-intensity or ≥150 min/week of moderate-intensity physical activity.

^h^≥150 min/week of vigorous-intensity or ≥300 min/week of moderate-intensity physical activity.

^i^Duration in hours/day.

^j^Coefficient of multiple determination.

## Discussion

The current study extends the literature on life satisfaction and PA by separately examining domain andintensity-specific PA levels. Our results indicated a weak positive relationship between leisure-time vigorous-intensity PA and life satisfaction among university students. For the other domain and intensity-specific types of PA, as well as for meeting MVPA recommendations, total PA, overall intensity-specific, and overall domain-specific PA levels we did not find significant independent associations with life satisfaction. Furthermore, higher life satisfaction was reported by females, younger students, and those with higher disposable income and self-rated general health.

The association of being a female with higher life satisfaction was congruent with the results of some, but not all previous studies of university students. For example, Vaez et al. [80] and Zhang et al. [81] found higher life satisfaction among female students, whilst non-significant [82,83] or mostly opposite [14] associations were observed in other studies. Furthermore, a recent study [81] found higher life satisfaction among freshmen when compared to older students, which is in accordance with the inverse relationship between age and life satisfaction observed in our study. By contrast, in several other studies among university students, life satisfaction showed no significant relationship with age or year in school [80,82,83]. Further, Zhang et al. [81] found no association between life satisfaction and urban/rural dwelling. Interestingly, among Swedish male adults (18–64 years of age) levels of satisfaction with leisure and family life were lower in those from more densely populated communities, whilst no such association existed among their female peers [84]. Our study indicated no relationship between size of community and life satisfaction among university students. Furthermore, previous within-nation studies showed positive relationships between personal income and life satisfaction of similar magnitude as in the current study [85]. Associated findings were inconsistent among university students, indicating a positive [83] or non-significant [81] relationship between life satisfaction and socio-economic status. Our results indicated no association between smoking status and life satisfaction, which is not in accordance with previous findings. For instance, in a large sample of university students from 21 countries, Grant et al. [14] found that non-smokers were more likely to have higher life satisfaction when compared to smokers. This was also supported by Vaez et al. [80]. Previous findings about the link between alcohol consumption and life satisfaction among university students were also inconsistent. In one study, moderate frequency of alcohol intake (2–4 times a month or 2–3 times a week) was associated with higher life satisfaction, when compared to no consumption [80]. By contrast, Murphy et al. [86] found a negative relationship between alcohol consumption and life satisfaction among female and no relationship among male students. As in the current study, no significant relationship between these variables was observed by Grant et al. [14] and Parkerson et al. [87]. Furthermore, we found no linear relationship between continuous BMI and life satisfaction. A previous study found a significant U-shaped relationship between BMI and life satisfaction among male and no relationship among female university students. To allow for possible non-linearity of the relationship, in a subsequent analysis (data not shown) we entered BMI as a categorical predictor, but despite that, no significant associations were observed. By far the strongest relationship with life satisfaction in our sample was found for self-rated general health, which is consistent with a previous study [80]. These comparisons indicate that findings about the socio-demographic and lifestyle correlates of life satisfaction among university students were inconsistent across different studies. As suggested by Grant et al. [14], it may be that the discrepancies between findings from different studies indicate culture- and country-specific nature of life satisfaction and its determinants among university students. Furthermore, we found no significant interaction effects between gender and self-rated health or any of the socio-demographic and lifestyle variables when modeling life satisfaction.

In unadjusted regression models, the adherence to MVPA guidelines, total PA, overall vigorous-intensity PA, overall walking, and overall leisure-time PA showed significant associations with life satisfaction. However, after controlling for the potential confounding variables, all the associations became non-significant. Only the relationship between leisure-time vigorous-intensity PA and life satisfaction remained significant after the adjustments. The positive relationship observed between these variables is in accordance with the results of previous studies [27,39,44]. Earlier research has also shown a positive association between walking and life satisfaction in elderly [44,45]. However, our results do not support this association among university students. Furthermore, it has been shown that adult and elderly gardeners have higher life satisfaction than non-gardeners [64,65]. Non-significant correlations between moderate and vigorous-intensity yard chores and life satisfaction obtained in the present study indicate that such relationship may not exist in university students.

A previous study found no relationship between total PA, intensity-specific PA levels (walking, moderate-intensity and vigorous-intensity) and life satisfaction [88], which is congruent with our results from the adjusted regression analysis (Models 3–5). It seems important to consider analyzing domain and intensity-specific PA levels in future studies (for example, vigorous-intensity PA in work domain, moderate-intensity PA in work domain, and walking in work domain), as drawing conclusions about the relationship (or lack thereof) between PA and life satisfaction based on total PA levels only may be misleading. Similarly, several previous studies have reported a positive association between non-intensity-specific PA in leisure-time and life satisfaction [14,31,40]. However, our analysis of domain and intensity-specific PA levels (Model 7) indicated that leisure-time moderate-intensity PA and leisure-time walking are not significantly related to life satisfaction. It may, therefore, be that the positive relationship between overall leisure-time PA and life satisfaction in the previous studies occurred only due to its one component−leisure-time vigorous-intensity PA, whilst its other two components (moderate-intensity leisure-time PA and leisure-time walking) may have been unrelated to life satisfaction. Hence, reporting domain-specific, but non-intensity-specific findings (for example, for total PA in leisure-time) may potentially lead to false conclusions and should be taken with caution in future studies.

As stated previously, *activity theory* suggests that life satisfaction depends on the frequency of participation in given activities and the degree of intimacy associated with the activities [21]. We assumed that, among university students, the degree of intimacy associated with all leisure-time PA (vigorous-intensity, moderate-intensity and walking) is similarly high. No relationship between life satisfaction and leisure-time moderate-intensity PA and leisure-time walking was, therefore, an unexpected finding. It is possible that the degree of intimacy associated with leisure-time vigorous-intensity PA was higher than with the other two components of leisure-time PA, which would partially explain our results from the perspective of *activity theory*. Since our study did not assess the degree of intimacy associated with different types of PA, these assumptions should be tested in future research. Alternatively, as mentioned earlier *need theory* implies that life satisfaction is regulated by the ability of an individual to satisfy his/her needs [24]. We assumed that all components of leisure-time PA equally satisfy primary, instrumental and perceived needs of young people (for example, need for movement, health needs, need for social contacts). However, it might be that vigorous-intensity PA in leisure-time satisfies these needs better than the other two components of leisure-time PA, resulting in its closer relation with life satisfaction. Since our study did not assess the psychological, social and environmental context of PA, these assumptions need to be tested in future studies.

A range of positive psychological effects are associated with regular exercise, including reduced depressive and anxiety symptoms [89], and improved sleep quality [90], global self-esteem [91] and body image [92]. The literature also suggests that exercise improves mood and increases individual’s capacity to cope with psychological stressors [93]. Furthermore, we assume that social environments might also play an important role in the relationship between leisure-time PA and life satisfaction. That is, exercising in social settings typically provides opportunities to interact with others which may be associated with access to emotional and instrumental support [94–96]. It is plausible that these social benefits of exercise mediate the relationship between leisure-time vigorous-intensity PA and life satisfaction. However, the mediating role of the same factors may also be assumed for the link between leisure time moderate-intensity PA and life satisfaction. It is, therefore, somewhat surprising that these two types of PA show different relationships with life satisfaction. To better understand this finding, future studies should aim to examine possible mechanisms underlying the relationship between different domain and intensity-specific PA levels and life satisfaction.

According to Cohen [70], the relative effect size (as expressed in terms of the partial correlation) of the relationship between leisure-time vigorous-intensity PA and life satisfaction can be deemed small. In comparison, the relative effect sizes of the relationships between life satisfaction and age and disposable income were also classified as ‘small,’ whilst the relative effects sizes of its associations with gender and self-rated health can be classified as ‘medium’. Furthermore, the current PA guidelines suggest that at least 75 minutes/week of vigorous-intensity or 150 minutes/week of moderate-intensity PA are required to reap significant health benefits [51]. The unstandardized regression coefficient showed that participants achieving 75 minutes/week of leisure-time vigorous-intensity PA experienced on average 0.44% higher life satisfaction than those reporting zero minutes of such activity (Table 3). This indicates that even if a university student should meet PA recommendations exclusively by engaging in vigorous-intensity PA, it would not necessarily be associated with practically significant changes in his/her life satisfaction.

Our findings do not support the utility of MVPA recommendations for increasing life satisfaction among university students. It may be that promoting exclusively leisure-time vigorous-intensity PA would be a more efficient approach to increase psychological well-being in this population. Taking into account that more than 50% of students do not engage in any leisure-time vigorous-intensity PA, it seems there is room for improvements. This should, however, be confirmed in future intervention research, as our cross-sectional study design did not allow us to test these assumptions.

The major strengths of the study were: 1) separate reporting of the relationships between 11 domain and intensity-specific PA levels and life satisfaction, which has not been done in previous studies; 2) use of a large random sample of university students; and 3) analyses adjusting for a wide range of common socio-demographic, lifestyle, and health-related covariates.

This study also has several limitations. First, cross-sectional data did not allow us to draw conclusions about the causality of the observed relationship. Second, previous studies have indicated that genetic factors influencing both frequency of exercise and life satisfaction may significantly mediate their relationship [40,97]. The design of our study did not allow us to examine the role of genetic factors. Third, PA levels were assessed using a self-reported questionnaire. A significant amount of random error in self-reports of PA might have affected our results by lowering the observed, when compared to true relationships, thus increasing the probability of type II error. Besides, it seems that the participants who reported engaging in physical activities were highly active (e.g., the median level of total physical activity was 780 minutes/week). A recent review has shown that self-reported physical activity levels are sometimes overestimated [98], which may partially explain this finding. Despite that, IPAQ-long form is an internationally comparable measure that has been widely used in epidemiological research. Moreover, contemporary objective measures of PA do not allow for calculation of domain-specific PA levels, which makes them unsuitable for this type of study [99]. Furthermore, in their recent studies, Maher et al. [36,37] investigated whether the influence of PA on daily life satisfaction is trait-like (i.e. time-invariant or top-down influence) or state-like (i.e. time-varying or bottom-up influence). Both studies indicated that daily life satisfaction is influenced by daily levels of PA rather than trait level of PA over longer periods of time. The current study only examined trait-like associations between PA and life satisfaction. It may be that the respective state-like associations would be different in magnitude and direction. Furthermore, the findings of the current study are only generalizable to the overall population of students, as no separate analyses were conducted for specific academic disciplines/degrees. Previous studies have shown that PA levels may significantly vary across different seasons [100,101]. Our survey took place in June, which limits the generalizability of our results to the other seasons and months. Moreover, the data were collected during the exam period, which may have affected students’ habitual PA levels and, consequently, biased our results. Finally, no information was collected on the environmental and social context of PA, which would allow for an even more thorough insight into its relationship with life satisfaction.

## Conclusions

The current study indicated a weak positive relationship between leisure-time vigorous-intensity PA and life satisfaction, whilst the other domain and intensity-specific types of PA, as well as meeting MVPA recommendations, total PA, overall intensity-specific, and overall domain-specific PA levels were not significantly associated with life satisfaction. We also found that higher life satisfaction is reported by females, younger students, and those with higher disposable income and self-rated general health. Our findings also underscore the importance of measuring and analyzing domain and intensity-specific PA levels in future studies, as drawing conclusions about the relationship between PA and life satisfaction based on total PA levels only may be misleading.

## Supporting Information

S1 Table Descriptive statistics for participants that reported at least some physical activity. ^a^Number (percentage) of participants that reported at least some physical activity. ^b^Median (interquartile range).
(DOCX)
