# Supplementary material for: Are Total, Intensity- and Domain-Specific Physical Activity Levels Associated with Life Satisfaction among University Students?
Source: PLoS One. 2015 Feb 19;10(2):e0118137. doi: 10.1371/journal.pone.0118137 (PMC4335071; doi:10.1371/journal.pone.0118137)
Supplement: S1 Table — aNumber (percentage) of participants that reported at least some physical activity. bMedian (interquartile range). (DOCX) [file pone.0118137.s001.docx]

**Table S1. Descriptive statistics for participants that reported at least some physical activity.**

| **Variable / category** | **n (%) non-zero**^a^ | **Median (IQR)**^b^ |
| --- | --- | --- |
| Physical activity type (minutes/week) |  |  |
| Vigorous-intensity at work | 83 (7.1) | 210 (270) |
| Moderate-intensity at work | 100 (8.6) | 240 (480) |
| Walking at work | 129 (11.1) | 240 (390) |
| Cycling for transport | 151 (13) | 120 (240) |
| Walking for transport | 1040 (89.4) | 240 (300) |
| Vigorous-intensity yard chores | 191 (16.4) | 150 (270) |
| Moderate-intensity yard chores | 612 (52.6) | 120 (180) |
| Moderate-intensity house chores | 822 (70.7) | 90 (150) |
| Vigorous-intensity in leisure-time | 521 (44.8) | 120 (190) |
| Moderate-intensity in leisure-time | 324 (27.9) | 120 (150) |
| Walking in leisure-time | 816 (70.2) | 180 (210) |
| Work domain | 173 (14.9) | 450 (780) |
| Transport domain | 1054 (90.6) | 280 (340) |
| Domestic domain | 898 (77.2) | 180 (340) |
| Leisure-time domain | 955 (82.1) | 270 (360) |
| Vigorous-intensity | 564 (48.5) | 150 (210) |
| Moderate-intensity | 981 (84.4) | 270 (450) |
| Walking | 1112 (95.6) | 418 (628) |
| Total (minutes/week) | 1137 (97.8) | 795 (1035) |
| Total (MET-minutes/week) | 1137 (97.8) | 3246 (4074) |
